# Flavonoids as CYP3A4 Inhibitors In Vitro

**DOI:** 10.3390/biomedicines12030644

**Published:** 2024-03-13

**Authors:** Martin Kondža, Ivica Brizić, Stela Jokić

**Affiliations:** 1 Faculty of Pharmacy, University of Mostar, Matice hrvatske bb, 88000 Mostar, Bosnia and Herzegovina; ivica.brizic@mef.sum.ba; 2 University Clinical Hospital Mostar, Kralja Tvrtka bb, 88000 Mostar, Bosnia and Herzegovina; 3 Faculty of Food Technology, Josip Juraj Strossmayer University of Osijek, 31000 Osijek, Croatia; sjokic@ptfos.hr

**Keywords:** CYP enzymes, CYP3A4, flavonoid, inhibition, natural products

## Abstract

Flavonoids, a diverse group of polyphenolic compounds found abundantly in fruits, vegetables, and beverages like tea and wine, offer a plethora of health benefits. However, they have a potential interaction with drug metabolism, particularly through the inhibition of the cytochrome P450 3A4 enzyme, the most versatile and abundant enzyme in the liver. CYP3A4 is responsible for metabolizing approximately 50% of clinically prescribed drugs across diverse therapeutic classes, so these interactions have raised concerns about potential adverse effects. This review delves into the scientific evidence surrounding flavonoid-mediated CYP3A4 inhibition, exploring the inhibitory potential of investigated flavonoids and future implications. Kusehnol I, chrysin, leachianone A, and sophoraflavone G showed the largest inhibitory potentials and lowest *IC_50_* values. While the clinical significance of flavonoid-mediated CYP3A4 inhibition in dietary contexts is generally considered low due to moderate intake and complex interactions, it poses a potential concern for individuals consuming high doses of flavonoid supplements or concurrently taking medications metabolized by CYP3A4. This can lead to increased drug exposure, potentially triggering adverse reactions or reduced efficacy.

## 1. Introduction

Dietary supplements based on natural ingredients are often used to support general health and well-being, but it is important to be aware of potential interactions with medications. Natural ingredients in dietary supplements can influence the metabolism and absorption of drugs, leading to changes in the effectiveness or safety of prescribed therapy [1]. Certain herbal extracts may potentially enhance or diminish the effects of specific medications, causing multiple clinical manifestations and adverse events [2,3,4]. Individual variations in metabolism and health status also play a role in these interactions, so it is important to monitor adverse drug reactions (ADRs) because dietary supplements, often derived from plants and other natural sources, may contain active compounds that can interact with prescription medications. These interactions can lead to a range of ADRs, including reduced drug efficacy, increased drug toxicity, or even life-threatening conditions [5]. Understanding these interactions can help healthcare providers and patients make informed decisions about the use of supplements alongside medications.

Certain dietary supplements may enhance the effectiveness of certain medications, potentially leading to improved treatment outcomes. For instance, certain flavonoids, found in fruits and vegetables, can enhance the absorption and bioavailability of certain antibiotics [6,7,8,9,10]. Conversely, supplements may interfere with the metabolism of medications, reducing their effectiveness or increasing their side effects. By understanding the potential interactions between supplements and medications, healthcare providers can tailor treatment plans to minimize the risk of ADRs and optimize the efficacy of both supplements and medications. This can lead to improved patient outcomes and overall health. Patients, on the other hand, should be aware of the potential interactions between the supplements and medications they are taking. By providing clear information about these interactions, healthcare providers can empower patients to make informed decisions about their healthcare and avoid potential risks.

Thoroughly understanding supplement–medication interactions is essential for safeguarding public health. By identifying and preventing potential ADRs, healthcare professionals can protect patients from harm and ensure the safe and effective use of both supplements and medications. This can lead to improved patient outcomes and overall health. The intricate interplay of hepatic cytochrome P450 (CYP) enzymes plays a significant role in modulating the pharmacokinetics of administered medications. Enzyme inhibition is a more prevalent phenomenon than induction [11]. Therefore, comprehending the mechanisms underlying enzyme inhibition and induction is crucial for optimizing the efficacy and safety of therapies with supplements or herbal medicines.

In order to determine the clinical significance and therapeutic manifestation of interactions between flavonoids and cytochrome P450 3A4 (CYP3A4) enzymes, the aim of this work was to provide an overview of the current knowledge in the field of inhibition of CYP3A4 enzymes by different groups of flavonoids. The review paper was made by searching the *PubMed* database for papers published with the keywords “CYP3A4; flavonoid; inhibition; in vitro” for the period until October 1st, 2023. The search resulted in 343 papers. After the initial analysis and proof-reading of abstracts, 87 papers were selected for further analysis. After reading and analyzing the papers, 29 papers were selected for research. The inclusion criteria were that the paper had to provide a specific flavonoid, flavonoid derivate, or flavonoid conjugate and had to provide in vitro inhibition details on CYP3A4 in the form of *IC_50_* and/or enzyme residual activity. A total of 86 flavonoids and their inhibitory potential toward CYP3A4 were explored. To the knowledge of the authors, this represents the biggest review of flavonoids and CYP3A4 inhibition. 

## 2. CYP Enzymes

CYP enzymes are a group of heme-containing enzymes that play important roles in the metabolism of many drugs and other xenobiotics. They are located in the endoplasmic reticulum of cells throughout the body, but they are most abundant in the liver [12]. CYP enzymes can catalyze a wide variety of reactions, including oxidation [13], reduction [14], hydrolysis [15], and isomerization [16]. The most common reaction catalyzed by CYP enzymes is oxidation. This leads to the molecule being more water soluble and easier to excrete from the body, but it can also make it more reactive and potentially toxic. Moreover, CYPs are involved in more than 90% of the reported enzymatic reactions [14].

**Figure 1:**
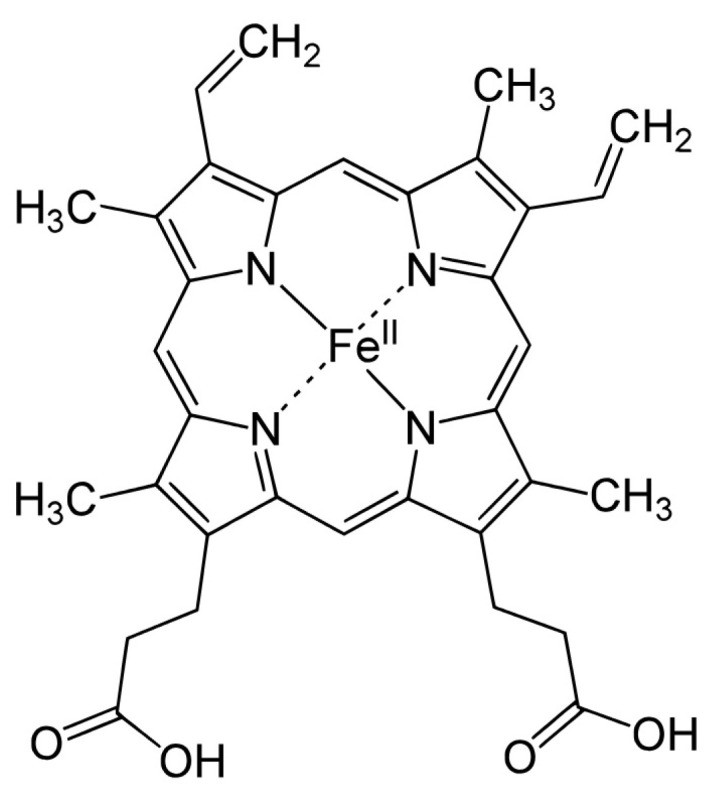
Hem structure formula.

CYP enzymes contain between 400 and 500 amino acid residues and one heme prosthetic group in the active site, iron in protoporphyrin IX [17]. In this structure, four pyrrole rings (I–IV) are interconnected by methyl bridges α, β, γ, and δ. Iron in the trivalent (ferric, Fe^3+^) form is located in the center of the protoporphyrin ring (Figure 1) and is coordinated by pyrrolic nitrogen. In addition, a water molecule is bound to the iron in the native structure. The heme iron is bound to the apoprotein via the thiol group of the cysteine residue. These are also the places for potential CYP inactivation by a covalent heme modification, by the modification of the apoprotein or by forming a pseudo-irreversible complex with iron [18]. 

CYP enzymes are part of a superfamily of enzymes that is further divided into 18 families, 43 subfamilies, and at least 57 different enzymes present in humans [15]. The division of the nomenclature of CYP enzymes is based on the similarity of their primary structure, or protein sequence [17], as shown in Table 1. The enzymes are encoded by a family of genes in the CYP superfamily. The specific CYP enzymes that are expressed in a particular cell or tissue depend on the genes that are present in that cell or tissue.

The role of these enzymes in the body is versatile. As mentioned earlier, these enzymes are not only present in the liver but also in the kidney, placenta, adrenal gland, gastrointestinal tract, and skin [19]. Thanks to their distribution throughout the body and the possibility of catalyzing a large number of different chemical reactions, CYP enzymes are responsible, among other things, for drug metabolism, steroid metabolism, bile acid biosynthesis, steroid biosynthesis, vitamin D deactivation, and much more [15]. This role of theirs extends doubly to their functions in human health and disease. CYP enzymes are responsible for numerous protective roles in the biotransformation of toxins and other harmful substances, as well as causing side effects and toxic elements through unproductive cycles of CYP enzymes [20].

An additional aspect of the importance of CYP enzymes lies in their role in antitumor therapy. CYP enzymes have been detected in tumor cells [21,22], where their expression is abnormal compared to the surrounding healthy tissue [23]. Accordingly, experts are actively working to use the CYP enzyme as a target in modeling oncology therapy, with CYP1B1 [24], CYP2J2 [25], and CYP2W1 [26] being extensively studied. The study of CYP enzymes is an important area of research in pharmacology, toxicology, and cancer biology. Understanding how CYP enzymes work can help design more effective and less toxic drugs and develop strategies for cancer prevention and treatment. 

### 2.1. CYP3A4 Enzyme

CYP3A4 is one of the most important enzymes involved in drug metabolism. It is encoded by the *CYP3A4* gene, located on chromosome 7q at the q21–22 locus, but variations in the coding of this gene are also responsible for variations in the presence of the CYP3A4 enzyme in humans [27]. It is not present in the fetus, but in most people, it is formed within a year of birth [28]. CYP3A4 is distributed in different tissues, but the highest presence of this enzyme, as well as the highest significance, was observed in the liver and intestine [29] and is responsible for more than 70% of gastrointestinal CYP activity [30]. CYP enzymes in the body catalyze more than 95% of oxidation and reduction reactions, while the CYP3A4 enzyme is responsible for catalyzing approximately 33% of such reactions [14]. It is believed that the large active site of this enzyme is responsible for participating in a large number of chemical reactions and, consequently, also in a large number of drug bio-transformations. The CYP3A4 enzyme is mentioned as the most important enzyme in drug metabolism, where it is considered to be involved in the metabolism of more than 50% of drugs [31]. Therefore, it is extremely important to know all the possible characteristics of this enzyme, especially the significantly present polymorphism of this enzyme. The rate of CYP3A4 metabolism can vary between individuals. This is a consequence of genetic polymorphisms, which can cause the enzyme to be more or less active. People with certain CYP3A4 polymorphisms may have a different rate of drug metabolism than people without these polymorphisms. For example, people with the CYP3A4*2C9 polymorphism have a higher risk of side effects from statins, which are metabolized by CYP3A4 [32]. In addition to drug dose adjustments, knowledge of CYP3A4 polymorphisms can help physicians identify people who are at higher risk of side effects. Some drugs can cause side effects if they are metabolized too quickly or too slowly. It is believed that there is 1- to 20-fold interindividual ‘variability’ of enzyme activity [30]. The levels of CYP3A4 in humans remain the same with increasing age; it is not influenced by external factors such as smoking or alcohol and is 25% more present in females [33].

When the CYP3A4 enzyme is mentioned, the CYP3A5 enzyme is often mentioned in the same context since it is an enzyme that is highly homologous and has overlapping substrates. Therefore, the term CYP3A4/5 enzyme is often used in the literature. However, it is important to emphasize that these two enzymes have different functions in some cases. For example, in the process of *O*^6^-demethylation of thebaine, the CYP3A5 isoform participates almost 10 times more than CYP3A4 [34]. The reaction marker for measuring CYP3A4 enzyme activity is the 6*β*-hydroxylation of testosterone. Nifedipine oxidation is used as well in order to further confirm the activity of the enzyme [17]. It has already been said that the CYP3A4 enzyme is involved in numerous chemical reactions (hydroxylation, aromatic oxidation, *N*- and *O*-dealkylation, etc.). Due to its large active site, it is able to both bind several substrates at once and create more complex metabolites through hydroxylation of the sp^3^ bond between carbon and hydrogen [35].

Many xenobiotics and endobiotics can act as CYP3A4 inducers, substrates, or inhibitors. The induction of CYP3A4 is less clinically significant than CYP3A4 inhibition, but it is necessary to understand because it can lead to decreased systematic exposure to co-administered drugs and result in inadequate therapeutic values of certain medications [36]. CYP3A4 induction occurs primarily at the transcriptional level, which involves the activation of the *CYP3A4* gene promoter, the region of DNA that regulates gene expression. Two major nuclear receptors, pregnane X receptor (PXR) and constitutive androstane receptor (CAR), are primarily responsible for CYP3A4 induction [37]. These receptors act as transcription factors, which means they bind to specific DNA sequences and recruit RNA polymerase, the enzyme responsible for DNA transcription. PXR is activated by a variety of compounds, including endogenous ligands such as bile acids and xenobiotics such as certain drugs, environmental pollutants, and pesticides. Upon binding to PXR, these ligands induce its translocation from the cytoplasm to the nucleus, where it binds to its heterodimeric partner, retinoid X receptor (RXRα), and interacts with specific DNA sequences in the *CYP3A4* gene promoter [38]. This interaction enhances the binding of RNA polymerase, leading to increased transcription of the *CYP3A4* gene and increased CYP3A4 protein expression. While previous models have assumed that enzyme induction is a rapid process driven by immediate changes in enzyme synthesis, more recent studies suggest that the induction response may be more complex and involve a lag phase before full induction is achieved. This delayed response could be attributed to the slower kinetics of mRNA synthesis, which may take several days to reach peak levels [39,40]. Some of the inducers of CYP3A4 include but are not limited to [41,42,43,44,45,46,47] apalutamide, capsaicin, carbamazepine, efavirenz, enzalutamide, modafinil, nevirapine, phenobarbital, phenytoin, rifampicin, St. John’s wort, and topiramate. Some of the substrates, on the other hand, include [48,49,50,51,52,53,54,55] aripiprazole, clarithromycin, cyclophosphamide, cyclosporin, doxorubicin, erythromycin, haloperidol, ifosfamide, ketoconazole, losartan, paclitaxel, sunitinib, tacrolimus, tamoxifen, verapamil, vincristine, and many others. 

### 2.2. CYP3A4 Inhibitors

Probably the most important item in the study of interactions of CYP enzymes with endobiotics and xenobiotics is the process of inhibition of CYP enzymes. Inhibitors are compounds that can bind to the active site or prevent the enzyme from catalyzing chemical reactions. In some cases, inhibitors can do both, which leads to a decrease in enzyme activity. A decrease in enzyme activity will consequently lead to a decrease in the biotransformation of substrates, in some cases of drugs, and may lead to an increased concentration of drugs in the blood system. Because of this, but also because of the influence on the development of new drugs, the processes of inhibition of various types of enzymes, including the CYP3A4 enzyme, are significantly studied. One of the most common criteria used to determine inhibitor strength is the 50% inhibitory concentration (*IC_50_*). The *IC_50_* value determines half of the maximum inhibitory concentration, a measure of the strength by which a certain compound can inhibit a biological or biochemical function [56]. *IC_50_* values are usually calculated using kinetic methods. One of the most common methods is the inhibition quadrant method. In this method, the enzyme is incubated with different concentrations of inhibitors, and the reaction rate is measured. The *IC_50_* value is then determined from the graph of the reaction rate in relation to the concentration of the inhibitor. A lower *IC_50_* value indicates a higher potency of the inhibitor. This means that a lower inhibitor concentration is required to achieve 50% inhibitory inactivation of the enzyme. According to the current literature guidelines on this topic, inhibitors are divided into strong, medium, and weak. Strong inhibitors are those that show an *IC_50_* value at a concentration of less than 1 μM, medium inhibitors are those that show an *IC_50_* value from 1 μM to 50 μM, and weak inhibitors are those that show an *IC_50_* value greater than 50 μM [57,58]. 

### 2.3. Types of CYP3A4 Inhibitions

When CYP3A4 enzyme inhibition is mentioned, it should be kept in mind that there are significantly different types of inhibition and, therefore, different clinical implications. CYP3A4 enzymes can be subject to reversible inhibition, in which the enzyme is bound by non-covalent bonds, which allows it to be easily removed from the enzyme and return to enzymatic activity. An example of a reversible inhibitor of the CYP3A4 enzyme is ketoconazole [59], which shows different types of inhibition—competitive and non-competitive inhibition. The third subtype of reversible inhibition, uncompetitive inhibition, is not a common case for CYP3A4 enzymes and is mentioned only sporadically [60]. A much more significant type of CYP3A4 enzyme inhibition is irreversible inhibition, in which the inhibitor is irreversibly bound to the enzyme by covalent bonds. Such a bond cannot be easily broken; therefore, the enzyme remains permanently inactive. One of the main characteristics of these inhibitions of the CYP3A4 enzyme is that it takes time; that is, it is a time-dependent inhibition [18]. 

As mentioned earlier, inhibition can be caused by the drug directly (or, in this case, by the flavonoid directly), or it can be caused by the metabolite that is produced by the CYP catalytic cycle [61]. An inhibition that is caused by the flavonoid directly can be classified as direct or time dependent. An inhibition that is caused by the metabolite can be classified as mechanism dependent (reversible or irreversible) or quasi-irreversible.

### 2.4. Methods for Testing out CYP3A4 Inhibitions

To test out the time-dependent type of inhibition, special experimental guidelines are implemented that suggest prior pre-incubation of the enzyme with the inhibitor to ensure sufficient time for enzyme inactivation. Only then is nicotinamide adenine dinucleotide phosphate (NADPH) added to the incubation mixture, which serves as a source of electrons in the case of testing CYP3A4 inhibition. NADPH is most often added in the form of a generating system. Another characteristic of this type of inhibition is that the *IC_50_* value cannot be reliably used as a basic indicator of inhibition potency, but other parameters must be considered [62]. Such inhibitions are not characteristic of CYP3A4 enzymes [63], but direct inhibition, as well as metabolism-dependent inhibition, are most often observed.

When testing direct inhibition, the generating system is immediately added to the incubation mixture, while for metabolism-dependent inhibition, pre-incubation with NADPH is carried out. Certain inhibitors of the CYP3A4 enzyme can also act in such a way as to show pseudo-irreversible inhibition. Pseudo-irreversible inhibition or quasi-irreversible inhibition occurs when the inhibitor binds to heme, that is, to the ferrous form of heme iron, whereby a stable complex is formed. Apparently, this type of inhibition should be considered irreversible. However, if there is a possibility for the same enzyme to return to its active form in in vitro conditions (for example, by using an oxidant along with dialysis), then one can observe this unusual phenomenon. One such example of an inhibitor is diltiazem [64]. Some of the selected CYP3A4 inhibitors and their mechanisms of inhibition (binding of the inhibitor to the protein and/or heme) are shown in Table 2.

Inhibition of the CYP3A4 enzyme must also be considered in a certain additional context. It is especially important to know on which material the inhibition was evaluated to better determine the environment in which the inhibition occurs. Tests of the inhibitory effect are most often conducted on the isolated CYP3A4 enzyme. This is a valuable approach that allows the use of an isolated enzyme to investigate inhibitory effects on CYP3A4. It allows researchers to control for a range of factors that may influence research results and to study inhibition at the molecular level. However, the inhibitory potential of the CYP3A4 enzyme is also often investigated on microsomes, which are obtained by centrifugation of tissue homogenates of mouse, rat, or human cells. Enzyme activity is often evaluated on human liver microsomes (HLMs), human intestinal microsomes (HIMs), and rat/mouse liver microsomes (RLM/MLMs). The advantage of this method is better enzyme activity due to binding to membranes [18,76]. To test CYP enzyme expression, the use of bacterial cells (*E. coli*) or baculovirus systems (baculosomes) obtained from insect cells is also considered. It is worth mentioning the use of hepatocytes for these purposes, especially if one wants to assess the toxicity of the effects of drugs in vitro [18].

## 3. Flavonoids

**Figure 2:**
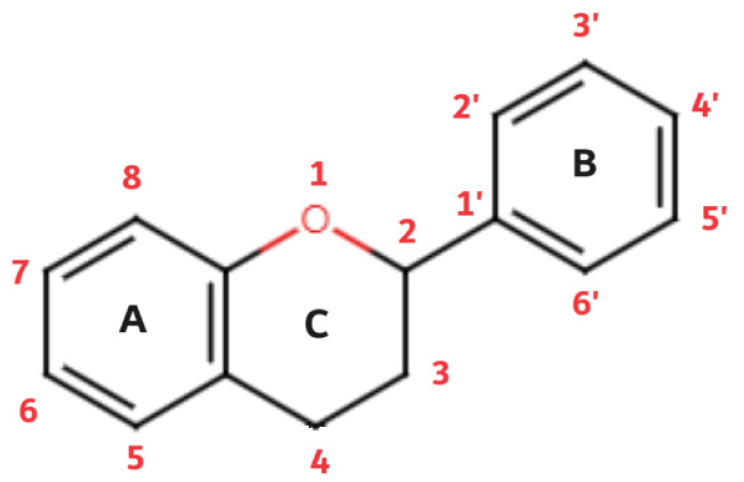
Flavonoid basic structure.

Flavonoids are a diverse group of plant pigments that exhibit a wide range of biological activities, making them of great interest to both scientists and the public alike. These naturally occurring compounds are found in a variety of fruits, vegetables, and beverages, and they are responsible for the vibrant colors of many plants [77]. The term *flavonoids* comes from the Latin word *flavus,* which means yellow; this refers to their characteristic of giving color to plants. The basic structure of flavonoids follows the polyphenolic concept; it consists of 15 carbon atoms that are connected to each other in a structure composed of three different rings (A, B, and C), of which two rings (A and B) are aromatic. The structure is connected in the form of a C6-C3-C6 group (Figure 2).

**Figure 3:**
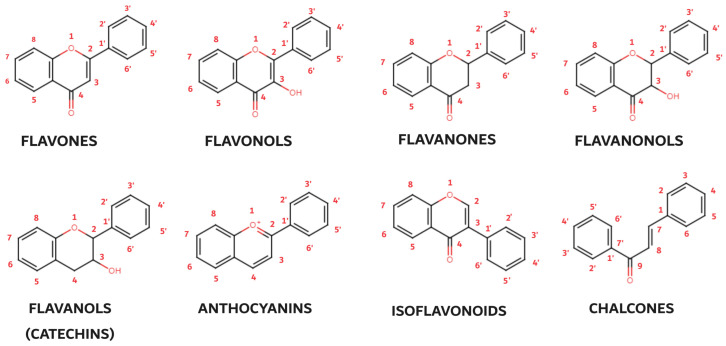
Basic structures of flavonoid subgroups.

Of the three benzene rings of the basic structure of flavonoids, rings A and C are mutually condensed, while ring C is substituted with an oxygen atom in position 1 (chromane ring). A benzene ring (B) is attached to the C2 position of the chromane ring. Depending on the basic structure of flavonoids, different groups and subgroups of flavonoids can be classified. This further division is based on different substituents on the basic structure of flavonoids, as well as on the degrees of oxidation and saturation of certain subgroups of flavonoids [78]. Thus, the following subgroups are most often described in the available literature: flavones, flavonols, flavanones, flavanonols, flavanols (catechins), anthocyanins, isoflavonoids, and chalcones (Figure 3).

Flavones, the simplest flavonoids, possess a double-bonded oxygen atom at the C4 position and a double bond between the C2 and C3 atoms. Flavonols, closely related to flavones, carry an additional hydroxyl group at the C3 position, enhancing their antioxidant and anti-inflammatory properties. Flavanones (dihydroflavones) lack the C2–C3 double bond, resulting in a more compact structure. Flavanonols, akin to flavanones, feature a hydroxyl group at the C3 position and a double-bonded oxygen atom at the C4 position. Catechins (flavonols), characterized by the presence of hydroxyl groups at the C3 position, are particularly abundant in green tea and other plants. Anthocyanins, the most colorful flavonoids, lack a substituted oxygen atom and a hydroxyl group, exhibiting a unique structural arrangement with double bonds at the O1–C2 and C3–C4 positions. Their protonated oxygen atom in position 1 contributes to their vibrant red, blue, or purple hues. Isoflavonoids, a special subgroup of flavonoids, exhibit a unique molecular arrangement with ring B attached to the C3 position rather than the C2 position as in other flavonoids. This structural deviation imparts specific biological activities to isoflavonoids, particularly their role in estrogen regulation. Chalcones, another flavonoid subgroup, deviate further from the typical flavonoid structure by lacking a ring C. The absence of the oxygen atom at position 1 disrupts the linkages between rings A and C, resulting in an open ring configuration. These diverse flavonoid subgroups, with their intricate structural variations, collectively contribute to the rich tapestry of plant pigments and their remarkable biological properties, ranging from antioxidant and anti-inflammatory effects to potential therapeutic applications.

Flavonoids are intricately synthesized through a multi-step process involving the condensation of acetate and shikimate pathway intermediates [79]. This intricate biosynthetic pathway, spanning several steps, orchestrates the transformation of simple starting materials into the complex structures of flavonoids. The initial step in flavonoid biosynthesis involves the condensation of *p*-coumaric acid, an acetate-derived intermediate, with 3-deoxy-*D*-arabinoheptulosonate-7-phosphate (DAHP), a shikimate pathway intermediate. This reaction is catalyzed by flavanone synthase (FLS), an enzyme that initiates the flavonoid branch of the shikimate pathway. The resulting intermediate, 4-coumaroyl-coenzyme A, undergoes further modifications, including hydroxylation, dehydration, and rearrangement reactions, leading to the formation of a series of intermediates with increasing complexity [80]. These intermediates serve as precursors for the synthesis of various flavonoid subgroups, including flavones, flavonols, flavanones, and anthocyanins [81]. The biosynthesis of flavonoids is regulated by a network of transcription factors, including MYB, WRKY, and bHLH factors, that respond to environmental cues [82]. These factors orchestrate the expression of flavonoid biosynthetic genes, ensuring that the plant produces the appropriate levels of these compounds to respond to the prevailing environmental conditions. Flavonoids play a multitude of roles in plant biology, contributing to their resilience and adaptability. Their involvement in plant defense against pathogens and herbivores is well documented [83,84,85]. Flavonoids can act as signaling molecules, attracting beneficial microbes or deterring pests. Additionally, they can directly interfere with pathogen growth or herbivore feeding mechanisms. Photosynthesis, the process by which plants convert light energy into chemical energy, is another area where flavonoids exhibit vital functions. Flavonoids act as antioxidants, protecting photosynthetic pigments from damage by reactive oxygen species (ROS) generated during photosynthesis. This protective role is crucial for maintaining the efficiency of photosynthesis and ensuring the overall health of the plant [86]. Moreover, flavonoids contribute to the plant’s overall antioxidant capacity, mitigating the harmful effects of ROS generated from various sources, including environmental stressors and metabolic processes. This antioxidant activity is attributed to the ability of flavonoids to scavenge ROS and neutralize their oxidizing potential, thereby protecting cellular components from damage. 

Flavonoids are ubiquitous in the plant kingdom, imparting a spectrum of colors ranging from yellow and orange to red and purple. Their presence is particularly pronounced in fruits, vegetables, and beverages, making them a significant component of the human diet [87]. So far, over 10,000 flavonoid compounds have been isolated and identified [88]. The distribution of flavonoids within plant tissues is highly variable, influenced by factors such as species, developmental stage, and environmental conditions. Generally, flavonoids are concentrated in the photosynthetic tissues, including leaves, fruits, and flowers [78]. This is due to their role in mediating plant responses to environmental stressors, such as ultra-violet (UV) radiation and pathogen attack. Fruits and vegetables are the primary sources of flavonoids in the human diet. Common dietary sources include berries, citrus fruits, apples, onions, grapes, tea, and red wine [89]. These foods contribute significantly to the daily intake of flavonoids, providing a rich source of antioxidants and potential health benefits.

Flavonoids have garnered considerable attention for their potential health benefits. These compounds play a crucial role in plant biology, serving as antioxidants and contributing to various physiological processes. Flavonoids exhibit a remarkable range of biological activities, including antiangiogenic [90], anticancer [91], antihypertensive [92], antimalarial [93], antimicrobial [94], antioxidant [93], antiproliferative [93], antitumor [93], cardioprotective [95], neuroprotective [96], and many other effects. The anti-inflammatory effects of flavonoids are attributed to their ability to modulate inflammatory signaling pathways. They can inhibit the production of pro-inflammatory mediators, such as cytokines and prostaglandins, and enhance the production of anti-inflammatory molecules, such as nitric oxide. This regulation of inflammation contributes to the prevention and management of chronic inflammatory conditions [97]. The anti-thrombotic properties of flavonoids are also noteworthy. They can inhibit the aggregation of platelets, the blood cells responsible for forming clots. This inhibition reduces the risk of blood clot formation, which can lead to stroke, heart attack, and other cardiovascular complications [98]. While more research is needed to fully elucidate the mechanisms underlying these benefits, accumulating evidence suggests that flavonoids play a protective role in various aspects of human health.

Humans are exposed to flavonoids via the vegetables and fruits they eat, the beverages they drink, and the diet supplements they take. The number of flavonoids in a food can vary depending on the variety of the food, how it is grown, and how it is processed. For example, strawberries that are grown in sunlight have more flavonoids than strawberries that are grown in shade. Flavonoids are also sensitive to heat and light. This means that they can break down when foods are cooked or stored in direct sunlight. The consumption of flavonoids and the exposure of people to flavonoids depends significantly from country to country and is dependent on the cultures of a particular society since it directly follows the eating habits of the population. In addition, it is very demanding to calculate the exact intake of flavonoids since the content of flavonoids in vegetables and fruits can vary significantly from region to region. However, there is some research on this topic. The global consumption of flavonoids ranges from 150 to 600 mg/day, depending on the region of the world [99]. For example, Hollman and Katan (1999) calculated that the average intake of flavones and flavonols in the Netherlands is approximately 23 mg/day. According to their claims, the main sources were tea, onions, and apples [100]. Although it is to be expected that Mediterranean countries will have a higher consumption of flavonoids, in the study conducted by Zamora-Ros et al. (2013) [101], an intake of 250 to 400 mg/day was determined, while in non-Mediterranean countries, an intake of 350 to 600 mg/day was determined. It is believed that the reason lies in the fact that the consumption of different types of tea is higher in non-Mediterranean countries. The average intake of flavonoids in the United States is 250 to 400 mg/day [102], while the average intake in Australia is much higher and amounts to 650 to 700 mg/day [103]. There are limited data from Asian countries; it is assumed that the average intake of flavonoids in China is up to 225 mg/day [104], and in South Korea, it is slightly higher (320 mg/day) [105]. All these indicators speak in favor of the fact that the intake of flavonoids in humans is diverse but certainly significant.

The mechanism of action of flavonoids by which they achieve different health effects is still extensively studied. One of the most frequently studied mechanisms of action is the inhibition of certain enzymes that can lead to certain pathological conditions. Thus, it is known that flavonoids can inhibit, for example, enzymes involved in cholesterol metabolism, such as 3-hydroxy-3-methylglutaryl coenzyme A reductase in Vero cells [106], enzymes involved in low-density lipoprotein oxidation such as 5-lipoxygenase [107], enzymes involved in inflammation and painful conditions such as cyclooxygenase 1 and 2 [108], or enzymes involved in tumor growth and proliferation [109]. The inhibitory effect of flavonoids on CYP enzymes, especially on the CYP3A4 enzyme [4,35,47,57], is certainly the most important for drug metabolism.

### 3.1. Flavones

Of all the tested flavonoids, most of them belong to the flavones group. In this group, 23 flavonoids were observed, for which inhibitory activity against CYP3A4 enzymes was tested. The average *IC_50_* value of the tested flavonoids was 38.36 μM, while the average remaining enzyme activity after incubation with flavonoids was 59.0%. In 40 reviewed experiments, it was observed that chrysin behaves as a strong inhibitor of the CYP3A4 enzyme, with an *IC_50_* value of 0.6 ± 0.5 μM in two different independent tests [110,111] (1, 43) (Table 3). A total of 25 flavones showed an intermediate inhibitory effect on the CYP3A4 enzyme, where certain experiments with much lower *IC_50_* values compared to the others should be highlighted. Apigenin has shown quite a low *IC_50_* value in several different tests. In the test conducted by Cho, Choi, and Burm, apigenin had an *IC_50_* value of 1.47 μM, where testosterone was used as a substrate, and the tests were performed on human liver microsomes [112]. When midazolam was used as a substrate for the CYP3A4 enzyme, this flavone showed an *IC_50_* value of 2.3 ± 0.3 μM [113]. Slightly higher values (8.4 μM) were obtained by Kondža et al. when nifedipine was used as a marker substrate on the CYP3A4 enzyme [110]. Shimada et al. (2010) tested numerous flavonoids and flavonoid derivatives on the CYP3A4 enzyme. They used midazolam as a marker substrate. The values obtained for 2’-methoxy-5,7-dihydroxyflavones and 3’4’-dimethoxy-5,7-dihydroxyflavones (2 and 6.5 μM, respectively) should be highlighted [113]. Luteolin also proved to be a potent inhibitor, which, in the test performed by Scott et al. [114], showed a significant *IC_50_* value of 4.62 ± 1.26 μM. Acacetin also proved to be a significant inhibitor of the enzyme in the Shimada et al. and Scott et al. assays. *IC_50_* values of 6.5 and 6.25 ± 0.96 μM were observed [113,114].

Tests of the remaining enzyme activity were performed on similar flavones using different concentrations of inhibitors (Table 4). Such results generally follow the results of *IC_50_* values. The effectiveness of acacetin, chrysin, and α-naphthoflavone should be singled out. The remaining enzyme activity in these cases was 5 ± 4%, 17 ± 3%, and 6.8% [115,116].

**Table 3:** Flavones as CYP3A4 inhibitors—*IC_50_* values.

Flavonoid	Substrate	Material	*IC_50_* (μM)	Potency [57]	Substituents									Ref.
					R_3_	R_5_	R_6_	R_7_	R_8_	R_2′_	R_3′_	R_4′_	R_5′_	
2′-methoxy-5,7-dihydroxyflavone	MDM	CYP3A4	2	intermediate	-	OH	-	OH	-	OCH_3_	-	-	-	[113]
3′4′-dimethoxy-5,7-dihydroxyflavone	MDM	CYP3A4	6.5	intermediate	-	OH	-	OH	-	-	OCH_3_	OCH_3_	-	[113]
3′-methoxy-5,7-dihydroxyflavone	MDM	CYP3A4	16	intermediate	-	OH	-	OH	-	-	OCH_3_	-	-	[113]
3-hydroxyflavone	TST	HLM	66.9 ± 4.0	weak	OH	-	-	-	-	-	-	-	-	[114]
5-hydroxyflavone	TST	HLM	103.82 ± 0.98	weak	-	OH	-	-	-	-	-	-	-	[114]
7-hydroxyflavone	TST	HLM	66.2 ± 5.8	weak	-	-	-	OH	-	-	-	-	-	[114]
acacetin	NFD	CYP3A4	7.5 ± 2.7	intermediate	-	OH	-	OH	-	-	-	OCH_3_	-	[110]
acacetin	TST	CYP3A4	10.9 ± 0.3	intermediate										[110]
acacetin	MDM	CYP3A4	6.5	intermediate										[113]
acacetin	TST	CYP3A4	10.9 ± 0.3	intermediate										[111]
acacetin	TST	HLM	6.25 ± 0.96	intermediate										[114]
apigenin	NFD	CYP3A4	8.4 ± 1.1	intermediate	-	OH	-	OH	-	-	-	OH	-	[110]
apigenin	TST	CYP3A4	11.4 ± 0.4	intermediate										[110]
apigenin	MDM	CYP3A4	2.3 ± 0.3	intermediate										[113]
apigenin	TST	CYP3A4	11.4 ± 0.4	intermediate										[111]
apigenin	TST	CYP3A4	30.8 ± 7.6	intermediate										[117]
apigenin	TST	HLM	1.47 ± 0.24	intermediate										[114]
baicalein	BTC	BAC	9.2	intermediate	-	OH	OH	OH	-	-	-	-	-	[112]
baicalein	TST	RLM	12.03	intermediate										[118]
baicalein	TST	HLM	9.60 ± 1.18	intermediate										[114]
chrysin	NFD	CYP3A4	2.5 ± 0.6	intermediate	-	OH	-	OH	-	-	-	-	-	[110]
chrysin	TST	CYP3A4	0.6 ± 0.5	strong										[110]
chrysin	QIN	HLM	70	weak										[119]
chrysin	MDM	CYP3A4	7.4 ± 1.1	intermediate										[113]
chrysin	TST	CYP3A4	0.6 ± 0.5	strong										[111]
chrysin	TST	CYP3A4	94.7 ± 30.9	weak										[117]
chrysin	TST	HLM	3.76 ± 1.13	intermediate										[114]
daidzein	TST	HLM	>100	weak	-	-	OH	-	-	-	-	OH	-	[114]
diosmetin	TST	HLM	58.6 ± 26.5	intermediate	-	OH	-	OH	-	-	OH	OCH_3_	-	[114]
flavone	QIN	HLM	224	weak	-	-	-	-	-	-	-	-	-	[119]
flavone	TST	HLM	>100	weak										[114]
luteolin	TST	CYP3A4	57.7 ± 16.1	weak	-	OH	-	OH	-	-	OH	OH	-	[117]
luteolin	TST	HLM	4.62 ± 1.26	intermediate										[114]
lysionotin	TST	HLM	13.85	intermediate	-	OH	OCH_3_	OH	OCH_3_	-	-	OCH_3_	-	[120]
nobiletin	TST	CYP3A4	20.6 ± 5.2	intermediate	-	OCH_3_	OCH_3_	OCH_3_	OCH_3_	-	-	OCH_3_	OCH_3_	[117]
scutellarin	MDM	HLM	>100	weak	-	OH	OH	O-GRD	-	-	-	OH	-	[121]
scutellarin	MDM	RLM	>100	weak										[121]
α-naphthoflavone 2′-propargyl ether	MDM	CYP3A4	64	weak	-	-	-	benz. R8	benz. R7	PGE	-	-	-	[113]
α-naphthoflavone 4′-propargyl ether	MDM	CYP3A4	55	weak	-	-	-	benz. R8	benz. R7	-	-	PGE	-	[113]
α-naphtoflavone	MDM	CYP3A4	18	intermediate	-	-	-	benz. R8	benz. R7	-	-	-	-	[113]

BAC—baculosome, benz. R7 and benz. R8—benzene ring condensed on the position R7–R8, BTC—7-benzyloxy-4-trifluoromethylcoumarine, HLM—human liver microsome, MDM—midazolam, NFD—nifedipin, O-GRD—*O*-glucuronide, PGE—propargyl ether, QIN—quinine, Ref.—reference, RLM—rat liver microsome, TST—testosterone.

When looking at the structure of the investigated flavonoids, a certain analogy can be observed in the relationship between structure and activity. Chrysin proved to be a strong inhibitor of the enzyme, and in relation to the basic structure, it has two hydroxyl functional groups at positions R_5_ and R_7_. Pang et al. (2017) [123] and Tiong et al. (2010) [124] determined that in addition to the molecular form and glycolization of the hydroxyl group, the specific number of hydroxyl groups is also responsible for the inhibitory effect of flavonoids. This was also confirmed in this research since it is evident that chrysin has distributed hydroxyl groups in the required positions. Apigenin has an additional hydroxyl group at the R_4’_ position, while luteolin has two additional hydroxyl groups at the R_3’_ and R_4’_ positions. Both of these flavonoids show a lower inhibitory activity compared to chrysin. For example, acacetin also proved to be quite a suitable inhibitor. Compared to chrysin, it has an additional methoxy group at the R_4’_ position. It is believed that the presence of this methoxy group makes acacetin a significantly stronger inhibitor compared to, for example, luteolin or apigenin [125].

In addition to in vitro studies, chrysin has been shown to be a potent inhibitor in in vivo studies. In a study published by Wang and Morris (2007), chrysin was administered simultaneously with the antibiotic nitrofurantoin [126]. Simultaneous administration of chrysin intraperitoneally at a concentration of 50 mg/kg and nitrofurantoin intravenously at a dose of 2 mg/kg was observed in comparison to the control (without flavonoids). A significant increase in AUC and C_max_ of nitrofurantoin by 1.76 and 1.72 times, respectively, was determined. Moreover, the cumulative hepatobiliary excretion of nitrofurantoin (1.5 mg/kg) was significantly reduced by approximately 75% after the coadministration of chrysin (50 mg/kg). Although this phenomenon is thought to occur due to the inhibition of breast cancer-resistant protein (BCRP/ABCG2) and less due to the inhibition of the CYP3A4 enzyme, this study highlights in detail the role of chrysin as a flavonoid with great biological potential and the need for caution during simultaneous use with drugs.

On the other hand, the inhibitory effect of baicalein on the CYP3A4 enzyme was further confirmed by in vivo studies. Cho et al. (2011) examined the effect of baicalein in doses from 0.4 to 8 mg/kg in rats on the pharmacokinetics and bioavailability of nimodipine (12 mg/kg) [112]. Baicalein at 8 mg/kg was found to increase C_max_ from 91 to 123 μg/L. The AUC of nimodipine increased from 509 to 587 μg/L x h. Both absolute and relative bioavailability increased by 3.4 and 15 value points. Meng et al. (2021) also examined baicalein in similar experimental settings in rat models but at a dose of 20 mg/kg [118]. They determined impaired values of simvastatin (40 mg/kg) and, more precisely, an increase in AUC and C_max_ by almost 220% and 103%, respectively. Such an increase in simvastatin concentration may result in serious side effects and clinical implications. Moreover, t_1/2_ increased from 4.89 to 10.18 h. All these findings confirm the transfer of the inhibitory effect from the in vitro environment to the in vivo environment.

### 3.2. Flavonols

In the group of flavonols, 12 different flavonoids were observed in 17 experimental studies. A review of flavonoids did not reveal a single strong CYP3A4 enzyme inhibitor according to current guidelines. A total of 14 flavonoids behaved as intermediate inhibitors of the CYP3A4 enzyme, while 3 flavonoids were weak inhibitors. The average *IC_50_* value of the observed inhibitors was 45.69 μM, while the average remaining enzyme activity was 58.99%. Of all observed flavonols, galangin had the lowest *IC_50_* value, which was 2.3 μM [113]. This test was performed on the CYP3A4 enzyme with midazolam as a substrate (Table 5). It is interesting to note that attention should be paid to the substrate that is applied and the material on which the test is carried out. Galangin was the subject of research by Ho et al., who in their work established an *IC_50_* value of galangin as high as 117 μM [119]. It should be taken into account that this research was conducted on human liver microsomes with quinine as a marker substrate. Valid literature for the CYP3A4 enzyme suggests the use of testosterone as a substrate marker with nifedipine as additional confirmation [18]. Chrysosplenetin also proved to be a significant inhibitor of the tested enzymes. An *IC_50_* value of 3.38 μM was observed. Midazolam was used as a marker substrate, and tests were performed on rat liver microsomes [127].

The remaining enzyme activity was tested with different inhibitor concentrations, from 1 μM to 188 μM (Table 6). Myricetin caused the greatest inhibition of the CYP3A4 enzyme, where at a concentration of 100 μM, it reduced the activity of the enzyme to 6.4% [119]. Kaempferitrin and dihydromyricetin, also at a concentration of 100 μM, caused a decrease in CYP3A4 enzyme activity to a value of 18% [128,129]. A similar inhibitory effect (18.27 ± 14.55%) was also demonstrated using chrysosplenetin (50 μM) [129].

**Table 5:** Flavonols as CYP3A4 inhibitors—*IC_50_* values.

Flavonoid	Substrate	Material	*IC_50_* (μM)	Potency [57]	Substituents									Ref.
					R_3_	R_5_	R_6_	R_7_	R_2′_	R_3′_	R_4′_	R_5′_	R_5′_	
chrysosplenetin	MDM	RLM	3.38	intermediate	-	OH	OCH_3_	OCH_3_	-	OCH_3_	-	-	-	[127]
dihydromyricetin	TST	HLM	14.75	intermediate	s.b.R_2_	OH	-	OH	-	OH	OH	OH	-	[129]
fisetin	QIN	HLM	44	intermediate	-	-	-	OH	-	-	OH	OH	-	[119]
fisetin	TST	CYP3A4	40.7 ± 7.4	intermediate										[117]
galangin	QIN	HLM	117	weak	-	OH	-	OH	-	-	-	-	-	[119]
galangin	MDM	CYP3A4	2.3	intermediate										[113]
kaempferitrin	TST	HLM	13.87	intermediate	R	OH	-	O-R	-	-	OH	-	-	[128]
kaempferol	QIN	HLM	223	weak	-	OH	-	OH	-	-	OH	-	-	[119]
kaempferol	TST	CYP3A4	32.65 ± 1.32	intermediate										[130]
kaempferol	TST	CYP3A4	18.3 ± 5.3	intermediate										[117]
kaempferol	TST	HLM	6.51 ± 1.01	intermediate										[114]
morin	QIN	HLM	75.2 ± 8.7	weak	-	OH	-	OH	OH	-	OH	-	-	[114]
myricetin	TST	HLM	10.7 ± 2.2	intermediate	-	OH	-	OH	-	OH	OH	OH	-	[114]
quercetin	TST	CYP3A4	28.0 ± 5.2	intermediate	-	OH	-	OH	-	OH	OH	-	-	[130]
quercetin	TST	CYP3A4	28.0 ± 5.2	intermediate										[117]
quercetin	MDM	RLM	27.1	intermediate										[131]
quercetin	TST	HLM	5.74 ± 1.16	intermediate										[114]

HLM—human liver microsome, RLM—rat liver microsome, MDM—midazolam, QIN—quinine, R—rhamnoside, Ref.—reference, TST—testosterone, s.b.R_2_—single bond with R_2_.

Again, the analogy of the structural arrangement of hydroxyl groups with the binding potential, i.e., the inhibitory potential of individual flavonoids, can be established. Galangin, which is structurally very similar to the previously mentioned chrysin, proved to be the most powerful inhibitor of this group. At positions R_5_ and R_7_, it has two hydroxyl groups, and its inhibitory potential is significantly stronger compared to other observed flavonoids in this group, which have more hydroxyl groups or methoxy groups as substituents. Although quercetin in the observed in vitro tests proved to be a weaker inhibitor compared to galangin (5.74 μM), there are in vivo studies that seriously approach the interaction potential of this flavonoid. Ahmadi et al. (2023) found that simultaneous administration of quercetin and amiodarone in rats leads to an increase in C_max_ and AUC_0-∞,_ which were elevated to 12.90% and 7.80%, respectively, while t_max_, t_1/2_, and CL declined by 16.70%, 2.35%, and 13.40% [134]. It has been shown that such simultaneous administration of quercetin (20 mg/kg) and amiodarone (50 mg/kg) can lead to serious clinical implications if the findings of this study are extrapolated to patients. Moreover, in an additional study, the authors found a similar pattern for warfarin [135]. Quercetin was administered to rats for 14 days, and on the 15th day, they received a single dose of warfarin. C_max_ of warfarin was increased by 30.43%, AUC_0-∞_ by 62.94%, and t_1/2_ by 10.54%, while CL decreased by 41.35% relative to the control.

### 3.3. Flavanones

In the group of flavanones, 35 different experiments were reviewed that examined the activity of 19 different flavonoids. The average *IC_50_* value of this group was 120.70 μM, while the average residual activity of the CYP3A4 enzyme was 61.54%. Kusehnol I, leachianone A, and sophoraflavone G proved to be the most powerful inhibitors [136]. These flavonoids showed a significant *IC_50_* value of 0.57, 0.69, and 0.78 μM (Table 7). At the same time, there are three strong inhibitors in this group. Ten weak inhibitors were observed in different experiments, while most were intermediate inhibitors. It is worth mentioning that other inhibitors from the subgroup of prenylated flavanones, such as kushenol M and kushenol C, also showed a significant *IC_50_* value (1.29 and 3.95 μM, respectively) on human liver microsomes with the use of midazolam [136]. Pinocembrin is a naturally occurring flavonoid that, in various studies, shows inhibitory activity against various enzymes, including CYP3A4 enzymes. In this review, similar *IC_50_* values of 4.30 to 5.00 μM were observed using midazolam as a substrate on human liver microsomes [110].

In the examination of the remaining activity of the CYP3A4 enzyme after the use of flavonoids as inhibitors, alpinetin proved to be the most potent inhibitor. At a concentration of 100 μM, it caused an inhibitory effect on CYP3A4, whereby the activity of this enzyme decreased to 20% on human liver microsomes [137] (Table 8). Pinocembrin, for example, showed a smaller effect. The remaining activity of the enzyme was approximately 50% [115]; however, the method of conducting the experiment and the incubation and pre-incubation processes should be taken into account in order to speak accurately about the inhibitory effect of this flavonoid.

**Table 7:** Flavanones as CYP3A4 inhibitors—*IC_50_* values.

Flavonoid	Substrate	Material	*IC_50_* (μM)	Potency [57]	Substituents										Ref.
					R_3_	R_5_	R_6_	R_7_	R_8_	R_2′_	R_3′_	R_4′_	R_5′_	R_6′_	
(*R*)-naringenin	TST	HLM	9.7	intermediate	-	OH	-	OH	-	-	-	OH	-	-	[138]
(*S*)-naringenin	TST	HLM	21.4	intermediate	-	OH	-	OH	-	-	-	OH	-	-	[138]
7-Hydroxyflavanone	TST	HLM	53.5 ± 7.2	weak	-	-	-	OH	-	-	-		-	-	[114]
alpinetin	TST	HLM	8.23	intermediate	-	OCH_3_	-	OH	-	-	-		-	-	[137]
eriodictyol	TST	HLM	65.2 ± 8.0	weak	-	OH	-	OH	-	-	OH	OH	-	-	[114]
flavanone	TST	HLM	101 ± 15	weak	-	-	-	-	-	-	-	-	-	-	[114]
hesperetin	QIN	HLM	163	weak	-	OH	-	OH	-	-	-	OCH_3_	OH	-	[119]
hesperetin	TST	HLM	61.8 ± 1.3	weak											[114]
hesperidin	QIN	HLM	601	weak	-	OH	-	O-G-R	-	-	OH	OCH_3_	-	-	[119]
kushenol A	MDM	HLM	>50	weak	-	OH	-	OH	MPH	-	-	-	-	OH	[136]
kushenol C	MDM	HLM	3.95	intermediate	-	OH	-	OH	MPH	OH	-	OH	-	-	[136]
kushebol I	MDM	HLM	0.57	strong	OH	OCH_3_	-	OH	MPH	OH	-	OH	-	-	[136]
kushenol M	MDM	HLM	1.29	intermediate	OH	OH	MP	OH	MPH	OH	-	OH	-	-	[136]
leachianone A	MDM	HLM	0.69	strong	-	OH		OH	MPH	-	-	OH	-	OCH_3_	[136]
naringenin	MDM	RLM	40.3	intermediate	-	OH	-	OH	-	-	-	OH	-	-	[131]
naringenin	QIN	HLM	139	weak											[119]
naringenin	TST	HLM	17.6	intermediate											[138]
naringenin	TST	HLM	6.50 ± 1.54	intermediate											[114]
naringin	QIN	HLM	1349	weak	-	OH		O-R-G	-	-	-	OH	-	-	[119]
neohesperidin	QIN	HLM	280	weak	-	OH		O-R-G	-	-	OH	OCH_3_	-	-	[119]
pinocembrin	NIF	CYP3A4	4.3 ± 1.1	intermediate	-	OH	-	OH	-	-	-	-	-	-	[110]
pinocembrin	TST	CYP3A4	5.0 ± 0.6	intermediate											[110]
pinocembrin	TST	CYP3A4	5.0 ± 0.6	intermediate											[111]
pinocembrin	TST	HLM	28.6 ± 6.1	intermediate											[114]
sophoraflavone G	MDM	HLM	0.78	strong	-	OH	-	O-G	-	-	-	OH	-	-	[136]

G—glucose, HLM—human liver microsome, MDM—midazolam, MP—2-methypent-2-ene, MPH—(2R)-5-methyl-2-(prop-1-en-2-yl)hex-4-en-1-yl, NIF—nifedipine, O-R-G—*O*-rhamnose-glucose, QIN—quinine, R—rhamnose, Ref.—reference, RLM—rat liver microsome, TST—testosterone.

### 3.4. Flavanonols, Flavanols/Catechins, Isoflavonoids, and Chalcones

Other observed groups of flavonoids are flavanonols, flavanols/catechins, isoflavonoids, and chalcones. The only tested flavonoid in the group of flavanonols was taxifolin. Taxifolin proved to be a weak inhibitor of CYP3A4, with an *IC_50_* value of 68.9 ± 2.3 μM [114] (Table 9). A total of eight different experiments and four different types of flavonoids were reviewed in the flavanols/catechins group. Most of them were intermediate potent flavonoids, with one, (−)-catechin-3-*O*-gallate, showing a weak inhibitory potential [139]. The average *IC_50_* value of this group was 37.22 μM, while the average residual value of the enzyme was 53% (Table 10). Examining the group of isoflavonoids, four different experimental processes and three different flavonoids were observed. The average *IC_50_* value was 43.73 μM. The most powerful inhibitor in this group was 8-methoxycirsilineol, with an *IC_50_* value of 1.71 ± 0.3 μM [117]. In the group of chalcones, three different experiments and three different flavonoids were observed. These are flavonoids of intermediate potential, with an average *IC_50_* value of 33.7 μM.

## 4. Conclusions

Flavonoids are a diverse group of phytochemicals found in fruits, vegetables, and beverages. They are responsible for the vibrant colors of many plants, as well as some of their unique flavors and aromas. Certain flavonoids, notably kusehnol I, chrysin, leachianone A, and sophoraflavone G, exhibit potent in vitro inhibitory effects on CYP3A4. While moderate dietary intake of these flavonoids typically poses a minimal risk due to complex interactions and lower individual concentrations, high-dose supplementation or concurrent use with CYP3A4-metabolized medications warrants closer examination. The inhibitory capacity of these flavonoids toward CYP3A4 can significantly elevate blood levels of co-administered drugs by reducing their metabolic clearance. This pharmacological interaction can manifest as increased adverse effects and reduced drug efficacy, among others. However, the clinical significance of this interaction in dietary contexts remains multifaceted. The interplay between various dietary constituents, individual metabolic capacities, and the specific flavonoid profile of different food sources complicates the prediction of in vivo effects. While dietary considerations are generally minor, high-dose flavonoid supplementation requires prudent evaluation alongside individual medication regimens involving CYP3A4 substrates to minimize potential adverse interactions. Physicians, pharmacists, health workers, and workers in the pharmaceutical and food industries should be aware of the possibilities of the strong and intermediate inhibitions shown in this review.

## Figures and Tables

**Table 1:** Nomenclature of CYP enzymes based on the similarity of the protein sequence.

Classification	Name	Amino Acid Homogeneity
Superfamily	CYP	-
Family	CYP3	≥40%
Subfamily	CYP3A	≥55%
Enzyme	CYP3A4	≥98%

**Table 2:** Selected drugs as CYP3A4 inhibitors.

Drug	Reversible Inhibitor	Mechanism of Inhibition	Reference
Amiodarone	Yes	Protein	[65]
Amlodipine	Yes	Heme	[66]
Boceprevir	No	Heme	[67]
Clarithromycin	No	Heme	[68]
Diclofenac	Yes	Protein	[69]
Diltiazem	Yes	Heme	[64]
Irinotecan	No	Protein	[70]
Levonorgestrel	Yes	Heme and protein	[71]
Oleandomycin	No	Heme	[72]
Prazosin	Yes	Protein	[73]
Ritonavir	Yes	Heme	[73]
Sertralin	Yes	Protein	[74]
Saquinavir	Yes	Protein	[75]

**Table 4:** Flavones as CYP3A4 inhibitors—enzyme residual activity.

Flavonoid	Substrate	Material	Conc. (μM)	CYP3A4 Residual Activity (%)	Substituents									Ref.
					R_3_	R_5_	R_6_	R_7_	R_8_	R_2′_	R_3′_	R_4′_	R_5′_	
acacetin	TST	BAC	100	5 ± 4	-	OH	-	OH	-	-	-	OCH_3_	-	[115]
apigenin	TST	BAC	100	24 ± 3	-	OH	-	OH	-	-	-	OH	-	[115]
apigenin	QIN	HLM	100	63.6										[119]
baicalein	BTC	BAC	10	50	-	OH	OH	OH	-	-	-	-	-	[112]
baicalein	TST	RLM	100	20										[118]
chrysin	TST	BAC	100	17 ± 3	-	OH	-	OH	-	-	-	-	-	[115]
chrysin	QIN	HLM	200	44.6										[119]
chrysin dimethylether	TST	BAC	100	61 ± 21	-	OCH_3_	-	OCH_3_	-	-	-	-	-	[115]
flavone	QIN	HLM	100	87.7	-	-	-	-	-	-	-	-	-	[119]
flavone	QIN	HLM	200	60.4										[119]
lysionotin	TST	HLM	100	13.67	-	OH	OCH_3_	OH	OCH_3_	-	-	OCH_3_	-	[120]
nobiletin	SAQM1	HLM	1	83.7 ± 4.1	-	OCH_3_	OCH_3_	OCH_3_	OCH_3_	-	-	OCH_3_	OCH_3_	[122]
nobiletin	SAQM3	HLM	1	85.2 ± 4.6										[122]
rutin	SAQM1	HLM	1	81.7 ± 1.4	O-G-R	OH	-	OH	-	-	OH	OH	-	[122]
rutin	SAQM3	HLM	1	83.0 ± 3.7										[122]
tangeretin	TST	BAC	1	42 ± 3	-	OCH_3_	OCH_3_	OCH_3_	OCH_3_	-	-	OCH_3_	-	[115]
tangeretin	SAQM1	HLM	1	89.0 ± 2.0										[122]
tangeretin	SAQM3	HLM	1	85.3 ± 6.1										[122]
α-naphtoflavone	TST	HLM	100	6.8	-	-	-	benz. R8	benz. R7	-	-	-	-	[116]

BAC—baculosome, benz. R7 and benz. R8—benzene ring condensed on the position R7–R8, BTC—7-benzyloxy-4-trifluoromethylcoumarine, conc.—concentration, HLM—human liver microsome, O-G-R—*O-*glucose-rhamnose, QIN—quinine, Ref.—reference, RLM—rat liver microsome, SAQM1—saquinavir (formation of M1 metabolite), SAQM3—saquinavir (formation of M1 metabolite), TST—testosterone.

**Table 6:** Flavonols as CYP3A4 inhibitors—enzyme residual activity.

Flavonoid	Substrate	Material	Conc. (μM)	CYP3A4 Residual Activity (%)	Substituents									Ref.
					R_3_	R_5_	R_6_	R_7_	R_8_	R_2′_	R_3′_	R_4′_	R_5′_	
astragalin	TST	CYP3A4	5	93.35	G	OH	-	OH	-	-	-	OH	-	[130]
chrysosplenetin	MDM	RLM	50	18.27 ± 14.55	-	OH	OCH_3_	OCH_3_	-	OCH_3_	-	-	-	[127]
dihydromyricetin	TST	HLM	100	18	-	OH	-	OH	-	OH	OH	OH	-	[129]
doxorubicine-quercetineconjugate	QIN	CYP3A4	100	50	-	OH	-	OH	-	-	OH	D	-	[132]
fisetin	QIN	HLM	100	27.5	-	-	-	OH	-	-	OH	OH	-	[119]
galangin	QIN	HLM	100	45.5	-	OH	-	OH	-	-	-	-	-	[119]
isoquercetin	TST	CYP3A4	5	83.32	G	OH	-	OH	-	-	OH	OH	-	[130]
isorahmetin	TST	BAC	100	73 ± 6	-	OH	-	OH	-	-	OCH_3_	OH	-	[115]
isorahmetin	BTC	PLM	128	60										[133]
kaempferitrin	TST	HLM	100	18	R	OH	-	O-R	-	-	OH	-	-	[128]
kaempferol	QIN	HLM	100	58.6	-	OH	-	OH	-	-	OH	-	-	[119]
kaempferol	TST	CYP3A4	5	73.8										[130]
morin	QIN	HLM	188	65.6	-	OH	-	OH	OH	-	OH	-	-	[119]
myricetin	QIN	HLM	100	6.4	-	OH	-	OH	-	OH	OH	OH	-	[119]
myricetin	BTC	PLM	128	60										[133]
quercetin	QIN	HLM	100	35.1	-	OH	-	OH	-	OH	OH	-	-	[119]
quercetin	TST	CYP3A4	5	66.82										[130]
quercetin	SAQ	HLM	1	90.6 ± 0.5										[122]

BAC—baculosome, BTC—7-benzyloxy-4-trifluoromethylcoumarine, conc.—concentration, D—doxorubicine, G—glucoside, HLM—human liver microsome, MDM—midazolam, PLM—pig liver microsome, QIN—quinine, R—rhamnoside, Ref.—reference, RLM—rat liver microsome, SAQ—saquinavir, TST—testosterone.

**Table 8:** Flavanones as CYP3A4 inhibitors—enzyme residual activity.

Flavonoid	Substrate	Material	Conc. (μM)	CYP3A4 Residual Activity (%)	Substituents										Ref.
					R_3_	R_5_	R_6_	R_7_	R_8_	R_2′_	R_3′_	R_4′_	R_5′_	R_6′_	
alpinetin	TST	HLM	100	20	-	OCH_3_	-	OH	-	-	-		-	-	[137]
hesperetin	QIN	HLM	100	64.5	-	OH	-	OH	-	-	-	OCH_3_	OH	-	[119]
hesperidin	QIN	HLM	100	79.6	-	OH	-	O-G-R	-	-	OH	OCH_3_	-	-	[119]
naringenin	QIN	HLM	100	60.9	-	OH	-	OH	-	-	-	OH	-	-	[119]
naringenin	SAQ	HLM	1	77.0 ± 1.1											[122]
naringin	QIN	HLM	100	51.1	-	OH		O-R-G	-	-	-	OH	-	-	[119]
naringin	SAQ	HLM	1	76.7 ± 0.3											[122]
neohesperidin	QIN	HLM	200	61.7	-	OH		O-R-G	-	-	OH	OCH_3_	-	-	[119]
pinocembrin	TST	BAC	100	50 ± 15	-	OH	-	OH	-	-	-	-	-	-	[115]
prunin	QIN	HLM	100	73.9		OH		O-G				OH			[119]

BAC—baculosome, G—glucose, HLM—human liver microsome, QIN—quinine, Ref.—reference, conc.—concentration, RLM—rat liver microsome, SAQ—saquinavir, TST—testosterone.

**Table 9:** Other flavonoids as CYP3A4 inhibitors—*IC_50_* values.

Flavonoid	Substrate	Material	*IC_50_* (μM)	Potency [57]	Substituents										Ref.
					R_2_	R_3_	R_4_	R_5_	R_6_	R_7_	R_8_	R_3′_	R_4′_	R_5′_	
**Flavanonols**															
taxifolin	TST	HLM	68.9 ± 2.3	weak	-	-	-	OH	-	OH	-	-	-	-	[114]
**Flavanols (Catechins)**															
(−)-catechin-3-*O*-gallate	MDM	HLM	71.5 ± 7.51	weak	-	GA	-	OH	-	OH	-	-	OH	OH	[139]
(−)-epigallocatechin-3-gallate	MDM	BAC	41	intermediate	-	GA	-	OH	-	OH	-	OH	OH	OH	[140]
(−)-epigallocatechin-3-gallate	MDM	HLM	23	intermediate											[140]
(−)-epigallocatechin-3-gallate	MDM	HLM	23.3	intermediate											[141]
(−)-epigallocatechin-3-*O*-gallate	MDM	HLM	23.7 ± 1.54	intermediate	-	O-GA	-	OH	-	OH	-	OH	OH	OH	[139]
(−)-epigallocatechin-3-*O*-gallate	MDM	HLM	40.8 ± 6.93	intermediate											[139]
**Isoflavonoids**															
7-hydroxy-isoflavone	NIF	HLM	129.5 ± 12.49	weak	-	-	-	-	-	OH	-	-	-	-	[142]
8-methoxycirsilineol	TST	CYP3A4	1.71 ± 0.3	intermediate	-	-	OH	OCH_3_	OCH_3_	OCH_3_	OH	-	-	OCH_3_	[117]
genistein	TST	HLM	>100	weak	-	-	-	OH	-	OH	-	-	OH	-	[114]
**Chalcones**															
TSAHC	MDM	HLM	>20	intermediate	-	-	OH	-	-	-	-	-	TSA	-	[143]
licochalcone A	MDM	HLM	47.4	intermediate	OCH_3_		OH	MBE					OH		[144]

BAC—baculosome, GA—gallic acid, HLM—human liver microsome, MBE—2-methylbut-3-en-2-yl, MDM—midazolam, NIF—nifedipine, Ref.—reference, TSA—4’-*p*-toluenesulfonylamide, TSAHC—4’-(*p*-toluenesulfonylamido)-4-hydroxychalcone, TST—testosterone.

**Table 10:** Other flavonoids as CYP3A4 inhibitors—enzyme residual activity.

Flavonoid	Substrate	Material	Conc. (μM)	CYP3A4 Residual Activity (%)	Substituents										Ref.
					R_2_	R_3_	R_4_	R_5_	R_6_	R_7_	R_8_	R_3′_	R_4′_	R_5′_	
**Flavanols (Catechins)**															
(-)-epicatechin	IRT	HLM	100	53	-	OH	-	OH	-	OH	-	-	OH	OH	[140]
(-)-epigallocatechin-3-gallate	IRT	HLM	100	53	-	GA	-	OH	-	OH	-	OH	OH	OH	[140]
**Isoflavonoids**															
7-hydroxy-isoflavone	NIF	HLM	100	75	-	-	-	-	-	OH	-	-	-	-	[142]
**Chalcones**															
N9	MDM	HLM	100	75	-	OCH_3_	-	-	OCH_3_	-	-	pyr.R_4′_	pyr.R_3′_	-	[143]

conc.—concentration, GA—gallic acid, HLM—human liver microsome, IRT—irinotecan, MDM—midazolam, N9—(2*E*)-1-(2,5-dimethoxyphenyl)-3-(6-quinoxalinyl)-2-propen-1-one, NIF—nifedipine, pyr. R4′ and pyr. R3′—pyrazine ring condensed on the position R4′—R3′, Ref.—reference.

## Data Availability

Not applicable.

## References

[1] Bailey D.G., Dresser G.K. (2004). Natural products and adverse drug interactions. CMAJ.

[2] Ruschitzka F., Meier P.J., Turina M., Luscher T.T., Noll G. (2000). Acute heart transplant rejection due to Saint John’s wort. Lancet.

[3] Piscitelli S.C., Burstein A.H., Chaitt D., Alfaro R.M., Falloon J. (2000). Indinavir concentrations and St John’s wort. Lancet.

[4] Kondža M., Mandić M., Ivančić I., Vladimir-Knežević S., Brizić I. (2023). *Artemisia annua* L. Extracts Irreversibly Inhibit the Activity of CYP2B6 and CYP3A4 Enzymes. Biomedicines.

[5] Choudhury A., Singh P.A., Bajwa N., Dash S., Bisht P. (2023). Pharmacovigilance of herbal medicines: Concerns and future prospects. J. Ethnopharmacol..

[6] Górniak I., Bartoszewski R., Króliczewski J. (2019). Comprehensive review of antimicrobial activities of plant flavonoids. Phytochem. Rev..

[7] Biharee A., Sharma A., Kumar A., Jaitak V. (2020). Antimicrobial flavonoids as a potential substitute for overcoming antimicrobial resistance. Fitoterapia.

[8] Donadio G., Mensitieri F., Santoro V., Parisi V., Bellone M., De Tommasi N., Izzo V., Piaz F.D. (2021). Interactions with Microbial Proteins Driving the Antibacterial Activity of Flavonoids. Pharmaceutics.

[9] Song M., Liu Y., Li T., Liu X., Hao Z., Ding S., Panichayupakaranant P., Zhu K., Shen J. (2021). Plant Natural Flavonoids Against Multidrug Resistant Pathogens. Adv. Sci..

[10] Ruddock P.S., Charland M., Ramirez S., López A., Towers G.H.N., Arnason J.T., Liao M., Dillon J.A.R. (2011). Antimicrobial Activity of Flavonoids from *Piper lanceaefolium* and Other Colombian Medicinal Plants against Antibiotic Susceptible and Resistant Strains of Neisseria gonorrhoeae. Sex. Transm. Dis..

[11] Bibi Z. (2008). Role of cytochrome P450 in drug interactions. Nutr. Metab..

[12] Manikandan P., Nagini S. (2018). Cytochrome P450 Structure, Function and Clinical Significance: A Review. Curr. Drug Targets.

[13] Coleman T., Podgorski M.N., Doyle M.L., Scaffidi-Muta J.M., Campbell E.C., Bruning J.B., De Voss J.J., Bell S.G. (2023). Cytochrome P450-catalyzed oxidation of halogen-containing substrates. J. Inorg. Biochem..

[14] Rendić S., Guengerich F.P. (2015). Survey of Human Oxidoreductases and Cytochrome P450 Enzymes Involved in the Metabolism of Xenobiotic and Natural Chemicals. Chem. Res. Toxicol..

[15] Zhao M., Ma J., Li M., Zhang Y., Jiang B., Zhao X., Huai C., Shen L., Zhang N., He L. (2021). Cytochrome P450 Enzymes and Drug Metabolism in Humans. Int. J. Mol. Sci..

[16] Gao L., Tu Y., Wegman P., Wingren S., Eriksson L.A. (2011). A mechanistic hypothesis for the cytochrome P450-catalyzed cis-trans isomerization of 4-hydroxytamoxifen: An unusual redox reaction. J. Chem. Inf. Model..

[17] Medić Šarić M., Rendić S., Medić-Šarić M. (2013). Cytochrome P450 enzymes. Metabolizam Lijekova i Odabranih Ksenobiotika.

[18] Bojić M. (2015). Predklinička ispitivanja inhibicijskog i interakcijskog potencijala novih lijekova na razini citokroma P450. Farm. Glas..

[19] Danielson P.B. (2002). The cytochrome P450 superfamily: Biochemistry, evolution and drug metabolism in humans. Curr. Drug Metab..

[20] Klyushova L.S., Perepechaeva M.L., Grishanova A.Y. (2022). The Role of CYP3A in Health and Disease. Biomedicines.

[21] Stipp M.C., Acco A. (2021). Involvement of cytochrome P450 enzymes in inflammation and cancer: A review. Cancer Chemother. Pharmacol..

[22] Burlaka V.S., Burlaka A.A. (2020). Cytochrome P450 content in primary tumors and liver metastases of patients with metastatic colorectal cancer. Exp. Oncol..

[23] Barros-Oliveira M.D.C., Costa-Silva D.R., Dos Santos A.R., Pereira R.O., Soares-Júnior J.M., Silva B.B.D. (2021). Influence of CYP19A1 gene expression levels in women with breast cancer: A systematic review of the literature. Clinics.

[24] Dutour R., Poirier D. (2017). Inhibitors of cytochrome P450 (CYP) 1B1. Eur. J. Med. Chem..

[25] Karkhanis A., Hong Y., Chan E.C.Y. (2017). Inhibition and inactivation of human CYP2J2: Implications in cardiac pathophysiology and opportunities in cancer therapy. Biochem. Pharmacol..

[26] Karlgren M., Ingelman-Sundberg M. (2007). Tumor-specific expression of CYP2W1: Its potential as a drug target in cancer therapy. Expert. Opin. Ther. Targets.

[27] Finta C., Zaphiropoulos P. (2000). The human cytochromeP450 3A locus. Gene evaluation by capture of downstream exons. Gene.

[28] Johnson T.N., Tucker G.T., Rostami-Hodjegan A. (2008). Development of CYP2D6 and CYP3A4 in the first year of life. CPT.

[29] Kudzi W., Dodoo A.N., Mills J.J. (2010). Genetic polymorphisms in MDR1, *CYP3A4* and *CYP3A5* genes in a Ghanaian population: A plausible explanation for altered metabolism of ivermectin in humans?. BMC Med. Genet..

[30] Shapiro L.E., Shear N.H. (2002). Drug interactions: Proteins, pumps, and P-450s. J. Am. Acad. Dermatol..

[31] Zanger U.M., Schwab M. (2013). Cytochrome P450 enzymes in drug metabolism: Regulation of gene expression, enzyme activities, and impact of genetic variation. Pharmacol. Ther..

[32] Hirota T., Fujita Y., Ieiri I. (2020). An updated review of pharmacokinetic drug interactions and pharmacogenetics of statins. Expert. Opin. Drug Metab. Toxicol..

[33] Hunt C.M., Westerkam W.R., Stave G.M. (1992). Effect of age and gender on the activity of human hepatic CYP3A4. Biochem. Pharmacol..

[34] Kramlinger V.M., Rojas M.A., Kanamori T., Guengerich F.P. (2015). Cytochrome P450 3A Enzymes Catalyze the O-6-Demethylation of Thebaine, a Key Step in Endogenous Mammalian Morphine Biosynthesis. JBC.

[35] Schmiedlin-Ren P., Edwards D.J., Fitzsimmons M.E., He K., Lown K.S., Woster P.M., Rahman A., Thummel K.E., Fisher J.M., Hollenberg P.F. (1997). Mechanisms of enhanced oral availability of CYP3A4 substrates by grapefruit constituents. Decreased enterocyte CYP3A4 concentration and mechanism-based inactivation by furanocoumarins. Drug Metab. Disp..

[36] Chu V., Einolf H.J., Evers R., Kumar G., Moore D., Ripp S., Silva J., Sinha V., Sinz M., Skerjanec A. (2009). In vitro and in vivo induction of cytochrome p450: A survey of the current practices and recommendations: A pharmaceutical research and manufacturers of america perspective. Drug Metab. Dispos..

[37] Gibson G.G., Plant N.J., Swales K.E., Ayrton A., El-Sankary W. (2002). Receptor-dependent transcriptional activation of cytochrome P4503A genes: Induction mechanisms, species differences and interindividual variation in man. Xenobiotica.

[38] Wang K., Chen S., Xie W., Wan Y.J. (2008). Retinoids induce cytochrome P450 3A4 through RXR/VDR-mediated pathway. Biochem. Pharmacol..

[39] Zhang J.G., Ho T., Callendrello A.L., Crespi C.L., Stresser D.M. (2010). A multi-endpoint evaluation of cytochrome P450 1A2, 2B6 and 3A4 induction response in human hepatocyte cultures after treatment with β-naphthoflavone, phenobarbital and rifampicin. Drug Metab. Lett..

[40] Pascussi J.M., Robert A., Nguyen M., Walrant-Debray O., Garabedian M. (2005). Possible involvement of pregnane X receptor-enhanced CYP24 expression in drug-induced osteomalacia. J. Clin. Investig..

[41] Gibbons J.A., de Vries M., Krauwinkel W., Ohtsu Y., Noukens J., van der Walt J.S., Mol R., Mordenti J., Ouatas T. (2015). Pharmacokinetic Drug Interaction Studies with Enzalutamide. Clin. Pharmacokinet..

[42] Johannessen S.I., Landmark C.J. (2010). Antiepileptic drug interactions - principles and clinical implications. Curr. Neupharmacol..

[43] Fuhr L.M., Marok F.Z., Hanke N., Selzer D., Lehr T. (2021). Pharmacokinetics of the CYP3A4 and CYP2B6 Inducer Carbamazepine and Its Drug-Drug Interaction Potential: A Physiologically Based Pharmacokinetic Modeling Approach. Pharmaceutics.

[44] Nallani S.C., Glauser T.A., Hariparsad N., Setchell K., Buckley D.J., Buckley A.R., Desai P.B. (2003). Dose-dependent induction of cytochrome P450 (CYP) 3A4 and activation of pregnane X receptor by topiramate. Epilepsia.

[45] Aquinos B.M., García Arabehety J., Canteros T.M., de Miguel V., Scibona P., Fainstein-Day P. (2021). Adrenal crisis associated with modafinil use. Medicina.

[46] Han E.H., Kim H.G., Choi J.H., Jang Y.J., Lee S.S., Kwon K.I., Kim E., Noh K., Jeong T.C., Hwang Y.P. (2012). Capsaicin induces CYP3A4 expression via pregnane X receptor and CCAAT/enhancer-binding protein β activation. Mol. Nutr. Food Res..

[47] Roby C.A., Anderson G.D., Kantor E., Dryer D.A., Burstein A.H. (2000). St John’s Wort: Effect on CYP3A4 activity. Clin. Pharmacol. Ther..

[48] Beunk L., Nijenhuis M., Soree B., de Boer-Veger N.J., Buunk A.M., Guchelaar H.J., Houwink E.J.F., Risselada A., Rongen G.A.P.J.M., van Schaik R.H.N. (2023). Dutch Pharmacogenetics Working Group (DPWG) guideline for the gene-drug interaction between CYP2D6, CYP3A4 and CYP1A2 and antipsychotics. Eur. J. Hum. Genet..

[49] Wen J., Chen Y., Zhao M., Hu W., Xiao Y.W. (2023). Effects of clarithromycin on the pharmacokinetics of tacrolimus and expression of CYP3A4 and P-glycoprotein in rats. Fundam. Clin. Pharmacol..

[50] Kumaraswami K., Katkam S.K., Aggarwal A., Sharma A., Manthri R., Kutala V.K., Rajasekhar L. (2017). Epistatic interactions among CYP2C19*2, CYP3A4 and GSTP1 on the cyclophosphamide therapy in lupus nephritis patients. Pharmacogenomics.

[51] Zhou S.F. (2008). Drugs behave as substrates, inhibitors and inducers of human cytochrome P450 3A4. Curr. Drug Metab..

[52] Bagdasaryan A.A., Chubarev V.N., Smolyarchuk E.A., Drozdov V.N., Krasnyuk I.I., Liu J., Fan R., Tse E., Shikh E.V., Sukocheva O.A. (2022). Pharmacogenetics of Drug Metabolism: The Role of Gene Polymorphism in the Regulation of Doxorubicin Safety and Efficacy. Cancers.

[53] Bansal S., Zamarripa C.A., Spindle T.R., Weerts E.M., Thummel K.E., Vandrey R., Paine M.F., Unadkat J.D. (2023). Evaluation of Cytochrome P450-Mediated Cannabinoid-Drug Interactions in Healthy Adult Participants. Clin. Pharmacol. Ther..

[54] Bilbao-Meseguer I., Jose B.S., Lopez-Gimenez L.R., Gil M.A., Serrano L., Castaño M., Sautua S., Basagoiti A.D., Belaustegui A., Baza B. (2015). Drug interactions with sunitinib. J. Oncol. Pharm. Pr. Pract..

[55] Ramos K.N., Gregornik D., Ramos K.S. (2021). Pharmacogenomics insights into precision pediatric oncology. Curr. Opin. Pediatr..

[56] Enting R.H., Hoetelmans R.M. (1998). Antiretroviral drugs and the central nervous system. AIDS.

[57] Shen G.L., Liang A.H., Zhao Y., Cao C.Y., Liu T., Li C.Y., Odd G.N. (2009). Interaction between four herb compounds and a western drug by CYP3A4 enzyme metabolism in vitro. China J. Chin. Mater. Med..

[58] Qin M.N., Liu R., Liu G.F., Dong F. (2012). Effects of Breviscapines Injections on CYP Activities in Rat Liver Microsomes in vitro. China Pharm..

[59] Greenblatt D.J., Zhao Y., Venkatakrishnan K., Duan S.X., Harmatz J.S., Parent S.J., Court M.H., von Moltke L.L. (2011). Mechanism of cytochrome P450-3A inhibition by ketoconazole. J. Pharm. Pharmacol..

[60] Deodhar M., Al Rihani S.B., Arwood M.J., Darakjian L., Dow P., Turgeon J., Michaud V. (2020). Mechanisms of CYP450 Inhibition: Understanding Drug-Drug Interactions Due to Mechanism-Based Inhibition in Clinical Practice. Pharmaceutics.

[61] A Guide to In Vitro CYP Inhibition Studies: Elements of Study Design and Important Considerations in Data Analysis. https://bioivt.com/resources/articles-publications/a-guide-to-in-vitro-cyp-inhibition-studies-elements-of-study-design-and-important-considerations-in-data-analysis.

[62] Fowler S., Zhang H. (2008). In vitro evaluation of reversible and irreversible cytochrome P450 inhibition: Current status on methodologies and their utility for predicting drug-drug interactions. AAPS J..

[63] Bojić M., Barbero L., Dolgos H., Freisleben A., Galleman D., Riva S., Guengerich F.P. (2014). Time- and NADPH-dependent inhibition of cytochrome P450 3A4 by the cyclopentapeptide cilengitide: Significance of the guanidine group and accompanying spectral changes. Drug Metab. Metabol. Disp..

[64] Lee J.Y., Lee S.Y., Oh S.J., Lee K.H., Jung Y.S., Kim S.K. (2012). Assesment of drug-drug interactions caused by metabolism-dependent cytochrome P450 inhibition. Chem. Biol. Interact..

[65] Polasek T.M., Elliot D.J., Lewis B.C., Miners J.O. (2000). An evaluation of potential mechanism-based inactivation of human drug metabolizing cytochromes P450 by monoamine oxidase inhibitors, including isoniazid. Br. J. Pharmacol. Exp. Ther..

[66] Jones D.R., Ekins S., Li L., Hall S.D. (2007). Computational approaches that predict metabolic intermediate complex formation with CYP3A4 (+b5). Drug Metab. Dispos..

[67] Hulskotte E.G., Feng H.P., Xuan F., Gupta S., van Zutven M.G., O’Mara E., Wagner J.A., Butterton J.R. (2013). Pharmacokinetic evaluation of the interaction between hepatitis C virus protease inhibitor boceprevir and 3-hydroxy-3-methylglutaryl coenzyme A reductase inhibitors atorvastatin and pravastatin. Antimicrob. Agents Chemother..

[68] Tinel M., Descatoire V., Larrey D., Loeper J., Labbe G., Letteron P., Pessayre D. (1989). Effects of clarithromycin on cytochrome P-450. Comparison with other macrolides. J. Pharmacol. Exp. Ther..

[69] Masubuchi Y., Ose A., Horie T. (2002). Diclofenac-Induced Inactivation of CYP3A4 and Its Stimulation by Quinidine. Drug Metab. Dispos..

[70] Hanioka N., Ozawa S., Jinno H., Tanaka-Kagawa T., Nishimura T., Ando M., Sawada J.I. (2002). Interaction of irinotecan (CPT-11) and its active metabolite 7-ethyl-10-hydroxycamptothecin (SN-38) with human cytochrome P450 enzymes. Drug Metab. Dispos..

[71] Guengerich F.P. (1990). Mechanism-based inactivation of human liver microsomal cytochrome P-450 IIIA4 by gestodene. Chem. Res. Toxicol..

[72] Iwata H., Tezuka Y., Kadota S., Hiratsuka A., Watabe T. (2005). Mechanism-based inactivation of human liver microsomal CYP3A4 by rutaecarpine and limonin from Evodia fruit extract. Drug Metab. Pharmacokinet..

[73] Watanabe A., Nakamura K., Okudaira N., Okazaki O., Sudo K.I. (2007). Risk assessment for drug-drug interaction caused by metabolism-based inhibition of CYP3A using automated in vitro assay systems and its application in the early drug discovery process. Drug Metab. Dispos..

[74] Von Moltke L.L., Durol A.L., Duan S.X., Greenblatt D.J. (2000). Potent mechanism-based inhibition of human CYP3A in vitro by amprenavir and ritonavir: Comparison with ketoconazole. Eur. J. Clin. Pharmacol..

[75] Ernest C.S., Hall S.D., Jones D.R. (2005). Mechanism-based inactivation of CYP3A by HIV protease inhibitors. J. Pharmacol. Exp. Ther..

[76] Guengerich F.P., Hayes A.W., Kruger C.L. (2014). Analysis and characterization of enzymes and nucleic acids relevant to toxicology. Hayes’ Principles and Methods of Toxicology.

[77] Shen N., Wang T., Gan Q., Liu S., Wang L., Jin B. (2022). Plant flavonoids: Classification, distribution, biosynthesis, and antioxidant activity. Food Chem..

[78] Panche A., Diwan A., Chandra S. (2016). Flavonoids: An overview. J. Nutr. Sci..

[79] Liu W., Feng Y., Yu S., Fan Z., Li X., Li J., Yin H. (2021). The Flavonoid Biosynthesis Network in Plants. Int. J. Mol. Sci..

[80] Liu Z.Q. (2022). What about the progress in the synthesis of flavonoid from 2020?. Eur. J. Med. Chem..

[81] Chen Y., Cheng F.B., Wu X.R., Zhu W., Liao J.W., Jiang Y., Zhang C., Niu W.Y., Yu Y., Duan H.Q. (2020). Flavonoid derivatives synthesis and anti-diabetic activities. Bioorg. Chem..

[82] Zhang J., Zhao H., Chen L., Lin J., Wang Z., Pan J., Yang F., Ni X., Wang Y., Wang Y. (2023). Multifaceted roles of WRKY transcription factors in abiotic stress and flavonoid biosynthesis. Front. Plant Sci..

[83] Mierziak J., Kostyn K., Kulma A. (2014). Flavonoids as important molecules of plant interactions with the environment. Molecules.

[84] Mathesius U. (2018). Flavonoid Functions in Plants and Their Interactions with Other Organisms. Plants.

[85] Ramaroson M.-L., Koutouan C., Helesbeux J.-J., Le Clerc V., Hamama L., Geoffriau E., Briard M. (2022). Role of Phenylpropanoids and Flavonoids in Plant Resistance to Pests and Diseases. Molecules.

[86] Brunetti C., Di Ferdinando M., Fini A., Pollastri S., Tattini M. (2013). Flavonoids as antioxidants and developmental regulators: Relative significance in plants and humans. Int. J. Mol. Sci..

[87] Thilakarathna S., Rupasinghe H. (2013). Flavonoid Bioavailability and Attempts for Bioavailability Enhancement. Nutrients.

[88] Kozlowska A., Szostak-Wegierek D. (2014). Flavonoids-food sources and health benefits. Rocz. Panstw. Zakl. Hig..

[89] Iwashina T. (2013). Flavonoid properties of five families newly incorporated into the order Caryophyllales (Review). Bull. Natl. Mus. Nat. Sci..

[90] Camero C.M., Germanò M.P., Rapisarda A., D’Angelo V., Amira S., Benchikh F., Braca A., De Leo M. (2018). Anti-angiogenic activity of iridoids from *Galium tunetanum*. Rev. Bras. Farm. Farmacogn..

[91] Zhao L., Yuan X., Wang J., Feng Y., Ji F., Li Z., Bian J. (2019). A review on flavones targeting serine/threonine protein kinases for potential anticancer drugs. Bioorganic Med. Chem..

[92] Xue Z., Wang J., Chen Z., Ma Q., Guo Q., Gao X., Chen H. (2018). Antioxidant, antihypertensive, and anticancer activities of the flavonoid fractions from green, oolong, and black tea infusion waste. J. Food Biochem..

[93] Patel K., Kumar V., Rahman M., Verma A., Patel D.K. (2018). New insights into the medicinal importance, physiological functions and bioanalytical aspects of an important bioactive compound of foods ‘Hyperin’: Health benefits of the past, the present, the future. Beni Suef Univ. J. Basic. Appl. Sci..

[94] Gupta T., Kataria R., Sardana S. (2022). A Comprehensive Review on Current Perspectives of Flavonoids as Antimicrobial Agent. Curr. Top. Med. Chem..

[95] Faggio C., Sureda A., Morabito S., Sanches-Silva A., Mocan A., Nabavi S.F., Nabavi S.M. (2017). Flavonoids and platelet aggregation: A brief review. Eur. J. Pharmacol..

[96] Xie J., Xiong J., Ding L.-S., Chen L., Zhou H., Liu L., Zhang Z.-F., Hu X.-M., Luo P., Qing L.-S. (2018). A efficient method to identify cardioprotective components of *Astragali Radix* using a combination of molecularly imprinted polymers-based knockout extract and activity evaluation. J. Chromatogr. A.

[97] Al-Khayri J.M., Sahana G.R., Nagella P., Joseph B.V., Alessa F.M., Al-Mssallem M.Q. (2022). Flavonoids as Potential Anti-Inflammatory Molecules: A Review. Molecules.

[98] Quintal Martínez J.P., Segura Campos M.R. (2023). Flavonoids as a therapeutical option for the treatment of thrombotic complications associated with COVID-19. Phytother. Res..

[99] An Overview of Global Flavonoid Intake and its Food Sources. https://www.intechopen.com/chapters/54289.

[100] Hollman P.C., Katan M.B. (1999). Dietary flavonoids: Intake, health effects and bioavailability. Food Chem. Toxicol..

[101] Zamora-Ros R., Knaze V., Luján-Barroso L., Romieu I., Scalbert A., Slimani N., Hjartåker A., Engeset D., Skeie G., Overvad K. (2013). Differences in dietary intakes, food sources and determinants of total flavonoids between mediterranean and non-mediterranean countries participating in the European Prospective Investigation into Cancer and Nutrition (EPIC) study. Br. J. Nutr..

[102] Chun O.K., Chung S.J., Song W.O. (2007). Estimated dietary flavonoid intake and major food sources of US adults. J. Nutr..

[103] Kent K., Charlton K.E., Russell J., Mitchell P., Flood V.M. (2015). Estimation of flavonoid intake in older Australians: Secondary Data Analysis of the Blue Mountains Eye Study. J. Nutr. Gerontol. Geriatr..

[104] Zhang Z., He L., Liu Y., Liu J., Su Y., Chen Y. (2014). Association between dietary intake of flavonoid and bone mineral density in middle aged and elderly Chinese women and men. Osteoporos. Int..

[105] Jun S., Shin S., Joung H. (2016). Estimation of dietary flavonoid intake and major food sources of Korean adults. Br. J. Nutr..

[106] Chen T.H., Liu J.C., Chang J.J., Tsai M.F., Hsieh M.H., Chan P. (2001). The in vitro inhibitory effect of flavonoid astilbin on 3-hydroxy-3-methylglutaryl coenzyme A reductase on Vero cells. Zhon Yi Xue Za Zhi.

[107] Schewe T., Kühn H., Sies H. (2002). Flavonoids of cocoa inhibit recombinant human 5-lipoxygenase. J. Nutr..

[108] Ribeiro D., Freitas M., Tomé S.M., Silva A.M., Laufer S., Lima J.L., Fernandes E. (2015). Flavonoids inhibit COX-1 and COX-2 enzymes and cytokine/chemokine production in human whole blood. Inflammation.

[109] Ferriola P.C., Cody V., Middleton E. (1989). Protein kinase C inhibition by plant flavonoids. Kinetic mechanisms and structure-activity relationships. Biochem. Pharmacol..

[110] Kondža M., Bojić M., Tomić I., Maleš Ž., Rezić V., Ćavar I. (2021). Characterization of the CYP3A4 Enzyme Inhibition Potential of Selected Flavonoids. Molecules.

[111] Kondža M., Rimac H., Maleš Ž., Turčić P., Ćavar I., Bojić M. (2020). Inhibitory Effect of Acacetin, Apigenin, Chrysin and Pinocembrin on Human Cytochrome P450 3A4. Croat. Chem. Acta.

[112] Cho Y.A., Choi J.S., Burm J.P. (2011). Effects of the antioxidant baicalein on the pharmacokinetics of nimodipine in rats: A possible role of P-glycoprotein and CYP3A4 inhibition by baicalein. Pharmacol. Rep..

[113] Shimada T., Tanaka K., Takenaka S., Murayama N., Martin M.V., Foroozesh M.K., Yamazaki H., Guengerich F.P., Komori M. (2010). Structure-function relationships of inhibition of human cytochromes P450 1A1, 1A2, 1B1, 2C9, and 3A4 by 33 flavonoid derivatives. Chem. Res. Toxicol..

[114] Scott L.M., Durant P., Leone-Kabler S., Wood C.E., Register T.C., Townsend A., Cline J.M. (2008). Effects of prior oral contraceptive use and soy isoflavonoids on estrogen-metabolizing cytochrome P450 enzymes. J. Steroid Biochem. Mol. Biol..

[115] Šarić Mustapić D., Debeljak Ž., Maleš Ž., Bojić M. (2018). The Inhibitory Effect of Flavonoid Aglycones on the Metabolic Activity of CYP3A4 Enzyme. Molecules.

[116] Niwa T., Toyota M., Kawasaki H., Ishii R., Sasaki S. (2021). Comparison of the Stimulatory and Inhibitory Effects of Steroid Hormones and α-Naphthoflavone on Steroid Hormone Hydroxylation Catalyzed by Human Cytochrome P450 3A Subfamilies. Biol. Pharm. Bull..

[117] Brahmi Z., Niwa H., Yamasato M., Shigeto S., Kusakari Y., Sugaya K., Onose J., Abe N. (2011). Effective cytochrome P450 (CYP) inhibitor isolated from thyme (*Thymus saturoides*) purchased from a Japanese market. Biosci. Biotechnol. Biochem..

[118] Meng M., Li X., Zhang X., Sun B. (2021). Baicalein inhibits the pharmacokinetics of simvastatin in rats via regulating the activity of CYP3A4. Pharm. Biol..

[119] Ho P.C., Saville D.J., Wanwimolruk S. (2001). Inhibition of human CYP3A4 activity by grapefruit flavonoids, furanocoumarins and related compounds. J. Pharm. Pharm. Sci..

[120] Li Y., Qin J., Wu H., Xu Y., Zhang L., Su K., Cui Y., Wang H. (2020). In vitro inhibitory effect of lysionotin on the activity of cytochrome P450 enzymes. Pharm. Biol..

[121] Han Y.L., Li D., Yang Q.J., Zhou Z.Y., Liu L.Y., Li B., Lu J., Guo C. (2014). In vitro inhibitory effects of scutellarin on six human/rat cytochrome P450 enzymes and P-glycoprotein. Molecules.

[122] Berginc K., Milisav I., Kristl A. (2010). Garlic flavonoids and organosulfur compounds: Impact on the hepatic pharmacokinetics of saquinavir and darunavir. Drug Metab. Pharmacokinet..

[123] Pang C.Y., Mak J.W., Ismail R., Ong C.E. (2012). In vitro modulatory effects of flavonoids on human cytochrome P450 2C8 (CYP2C8). Naunyn Schmiedebergs Arch. Pharmacol..

[124] Tiong K.H., Yiap B.C., Tan E.L., Ismail R., Ong C.E. (2010). In vitro modulation of naturally occurring flavonoids on cytochrome P450 2A6 (CYP2A6) activity. Xenobiotica.

[125] Šarić Mustapić D. (2020). Utjecaj flavonoida na metaboličku aktivnost enzima citokrom P450 u uvjetima in vitro. Ph.D. Thesis.

[126] Wang X., Morris M.E. (2007). Effects of the Flavonoid Chrysin on Nitrofurantoin Pharmacokinetics in Rats: Potential Involvement of ABCG2. Drug Metab. Dispos..

[127] Wei S., Ji H., Yang B., Ma L., Bei Z., Li X., Dang H., Yang X., Liu C., Wu X. (2015). Impact of chrysosplenetin on the pharmacokinetics and anti-malarial efficacy of artemisinin against *Plasmodium berghei* as well as in vitro CYP450 enzymatic activities in rat liver microsome. Malar. J..

[128] Zhang N., Liu J., Chen Z., Dou W. (2019). In vitro inhibitory effects of kaempferitrin on human liver cytochrome P450 enzymes. Pharm. Biol..

[129] Liu L., Sun S., Rui H., Li X. (2017). In vitro inhibitory effects of dihydromyricetin on human liver cytochrome P450 enzymes. Pharm. Biol..

[130] Zhang Z.J., Xia Z.Y., Wang J.M., Song X.T., Wie J.F., Kang W.Y. (2016). Effects of Flavonoids in *Lysimachia clethroides* Duby on the Activities of Cytochrome P450 CYP2E1 and CYP3A4 in Rat Liver Microsomes. Molecules.

[131] Pilipenko N., Ropstad E., Halsne R., Zamaratskaia G. (2017). Effect of Naringenin, Quercetin, and Sesamin on Xenobiotica-Metabolizing CYP1A and CYP3A in Mice Offspring after Maternal Exposure to Persistent Organic Pollutants. Biomed. Res. Int..

[132] Alrushaid S., Zhao Y., Sayre C.L., Maayah Z.H., Forrest M.L., Senadheera S.N., Chaboyer K., Anderson H.D., El-Kadi A.O.S., Davies N.M. (2017). Mechanistically elucidating the in vitro safety and efficacy of a novel doxorubicin derivative. Drug Deliv. Transl. Res..

[133] Ekstrand B., Rasmussen M.K., Woll F., Zlabek V., Zamaratskaia G. (2015). In vitro gender-dependent inhibition of porcine cytochrome p450 activity by selected flavonoids and phenolic acids. Biomed. Res. Int..

[134] Ahmad E., Janhangir M., Bukhari N.I., Khan J., Sarwar A., Aziz T., Nabi G., Alharbi M., Thamer H.A., Alasmari A.F. (2023). Influence of quercetin on amiodarone pharmacokinetics and biodistribution in rats. Eur. Rev. Med. Pharmacol. Sci..

[135] Ahmad E., Jahangir M., Ismail M.A., Afzal H., Bano S., Shamim R., Bukhari N.I. (2023). Influence of Quercetin Pretreatment on Pharmacokinetics of Warfarin in Rats. Curr. Drug Saf..

[136] Yim D., Kim M.J., Shin Y., Lee S.J., Shin J.G., Kim D.H. (2019). Inhibition of Cytochrome P450 Activities by *Sophora flavescens* Extract and Its Prenylated Flavonoids in Human Liver Microsomes. Evid. Based Complement. Altern. Med..

[137] Song H., Wei C., Yang W., Niu Z., Gong M., Hu H., Wang H. (2022). Alpinetin suppresses CYP3A4, 2C9, and 2E1 activity in vitro. Pharm. Biol..

[138] Lu W.J., Ferlito V., Xu C., Flockhart D.A., Caccamese S. (2011). Enantiomers of naringenin as pleiotropic, stereoselective inhibitors of cytochrome P450 isoforms. Chirality.

[139] Satoh T., Fujisawa H., Nakamura A., Takahashi N., Watanabe K. (2016). Inhibitory Effects of Eight Green Tea Catechins on Cytochrome P450 1A2, 2C9, 2D6, and 3A4 Activities. J. Pharm. Pharm. Sci..

[140] Albassam A.A., Markowitz J.S. (2017). An Appraisal of Drug-Drug Interactions with Green Tea (*Camellia sinensis*). Planta Med..

[141] Misaka S., Kawabe K., Onoue S., Werba J.P., Giroli M., Tamaki S., Kan T., Kimura J., Watanabe H., Yamada S. (2013). Effects of green tea catechins on cytochrome P450 2B6, 2C8, 2C19, 2D6 and 3A activities in human liver and intestinal microsomes. Drug Metab. Pharmacokinet..

[142] Monostory K., Vereczkey L., Lévai F., Szatmári I. (1998). Ipriflavone as an inhibitor of human cytochrome P450 enzymes. Br. J. Pharmacol..

[143] Im Y., Kim Y.W., Song I.S., Joo J., Shin J.H., Wu Z., Lee H.S., Park K.H., Liu K.H. (2012). Effect of TSHAC on human cytochrome P450 activity, and transport mediated by P-glycoprotein. J. Microbiol. Biotechnol..

[144] Li G., Simmler C., Chen L., Nikolić D., Chen S.N., Pauli G.F., van Breemen R.B. (2017). Cytochrome P450 inhibition by three licorice species and fourteen licorice constituents. Eur. J. Pharm. Sci..

